# Determination of Multiple Fluorescent Brighteners in Human Plasma Using Captiva EMR-Lipid Clean-Up and LC-MS/MS Analysis

**DOI:** 10.3390/toxics13050352

**Published:** 2025-04-28

**Authors:** Yubing Yan, Bowen Liang, Jiawen Yang, Qing Deng, Xiaoying Liang, Hui Chen, Bibai Du, Lixi Zeng

**Affiliations:** 1 Guangdong Key Laboratory of Environmental Pollution and Health, College of Environment and Climate, Jinan University, Guangzhou 511443, China; yubingyan0207@163.com (Y.Y.); liangbowen8867@163.com (B.L.); yangjw0803@163.com (J.Y.); 17340512415@163.com (Q.D.); liangxiaoying05@163.com (X.L.); lxzeng@jnu.edu.cn (L.Z.); 2 Guangdong Provincial Academy of Environmental Science, Guangzhou 510045, China; chenhui20003@163.com; 3 College of Resources and Environmental Engineering, Guizhou University, Guiyang 550025, China; 4 School of Resources and Environmental Science, Quanzhou Normal University, Quanzhou 362000, China

**Keywords:** new pollutant, fluorescent whitening agent, lipid removal, solid-phase extraction (SPE), human blood, human exposure

## Abstract

Fluorescent brighteners (FBs) are a class of chemicals extensively used in industrial and consumer products. Their environmental occurrences and potential health risks have raised significant concerns. However, the lack of analytical methods for FBs in human samples has hindered the accurate assessment of internal exposure levels. Addressing this gap, this study developed and validated a novel method for the simultaneous determination of 13 FBs at trace levels in human plasma using solid-phase extraction combined with HPLC-MS/MS. The method employed EMR-Lipid SPE columns, which can selectively adsorb phospholipids for plasma sample pre-treatment. Detection was achieved through positive ion electrospray ionization (ESI) in multiple reaction monitoring (MRM) modes. The results showed that all 13 FBs exhibited good linearity within their respective ranges, with correlation coefficients (R^2^) greater than 0.992. The method quantitation limits (MQLs) of the FBs ranged from 0.012 to 0.348 ng/mL, and the spiked recovery rates ranged from 61% to 98%. The method was successfully applied to analyze 10 adult plasma samples, detecting 10 FBs with total concentrations ranging from 0.221 to 0.684 ng/mL. This study provides a reliable analytical method for determining FBs in human plasma, providing a basis for further research on human internal exposure to FBs and associated health risk assessments.

## 1. Introduction

Fluorescent brighteners (FBs) are a class of chemicals that absorb ultraviolet (UV) light, which is imperceptible to the human eye, and re-emit it as blue or blue-violet fluorescence, thereby enhancing the perceived brightness and whiteness of materials [1]. Owing to their optical properties, FBs are extensively utilized in various industries, such as textiles, detergents, cosmetics, paper manufacturing, and food packaging coatings. They have also been illicitly added to food products such as flour and rice [2,3,4,5]. Currently, FBs are classified as high-production-volume chemicals, with thousands of known FB variants, encompassing both ionic and non-ionic structures, among which 40–50 types are commonly employed in commercial applications [6,7]. Existing toxicological studies have found that FBs may cause developmental toxicity and alter gene expression in zebrafish [8]. According to Gloxhuber et al., FBs can persist on human skin for extended periods, potentially causing irritation [9,10]. Moreover, repeated exposure of the skin to FBs combined with UV radiation has been associated with carcinogenesis in mice [11]. Certain FBs have been listed by the United States Environmental Protection Agency (USEPA) as persistent, bioaccumulative, and toxic (PBT) chemicals [12]. China has established a series of standards to regulate the permissible levels of FBs in consumer products [13,14].

The widespread use of FBs is expected to cause their substantial release into the environment. However, to date, most environmental studies on FBs were conducted prior to the year 2000, primarily focusing on two commonly used FBs, FB 351 and FB 71. Recently, our investigations positively detected 17 and 25 different FBs in urban indoor dust and municipal sludge, with median total concentrations of 11,000 ng/g and 35,300 ng/g, respectively [15,16]. These findings provide clear evidence that FBs have already caused significant environmental pollution. Currently, the extent of human exposure to FBs remains poorly understood. Given the widespread environmental presence and toxicological effects of FBs, there is an urgent need to develop qualitative and quantitative analytical methods to assess internal human exposure levels to FBs, which is essential for further evaluating their potential health risks.

For the detection of FBs, several analytical techniques are currently available, including ultraviolet–visible (UV–Vis) spectrophotometry, multimolecular infrared (MM-IR) spectroscopy coupled with stereomicroscopy, high-performance liquid chromatography with fluorescence detection (HPLC-FLD), and high-performance liquid chromatography-tandem mass spectrometry (HPLC-MS/MS) [4,5,17,18,19,20]. The detection limits of spectrophotometry and MM-IR spectroscopy combined with stereomicroscopy are typically higher than 1 μg/mL, and these methods are unsuitable for high-throughput analysis. Although HPLC-FLD can meet the requirements for high-throughput analysis, its detection limit is generally in the range of a few nanograms per milliliter, and its performance is significantly influenced by the fluorescent properties of the analytes. Considering the variability in fluorescent properties among different FBs, HPLC-FLD may not be suitable for trace analysis of multiple FBs in human samples. In contrast, HPLC-MS/MS enables high-throughput analysis with a detection limit below 1 ng/mL, regardless of the fluorescent properties of the analytes. In fact, we have successfully applied HPLC-MS/MS for the simultaneous analysis of multiple FBs in environmental samples [15,16]. Therefore, this method holds great potential for assessing the internal exposure levels of FBs in humans.

Blood is the most direct medium for monitoring human exposure to environmental chemicals. However, proteins and phospholipids in blood can significantly interfere with mass spectrometry analysis, making sample pretreatment methods previously developed for environmental samples potentially unsuitable. In this study, we developed a method for the determination of 13 FBs in human plasma using Captiva EMR-Lipid solid-phase extraction (SPE) cartridges for sample purification and HPLC-MS/MS analysis. This method was successfully applied to actual samples and demonstrated satisfactory validation parameters, including detection limits, linearity of calibration curves, recovery rates, and matrix effects, enabling trace analysis of multiple FBs in human plasma. This approach will provide an analytical strategy for subsequent large-scale population exposure assessments and health risk studies.

## 2. Materials and Methods

### 2.1. Chemicals and Reagents

This study focuses on the development of analytical methods for 13 FBs that are commonly used commercially, including KSN (FB 368), KCB (FB 367), SWN (FB 52/FB 140), PF (DT/FB 135), OB (FB 184), OB-1 (FB 393), OB-2, ER-I (FB 199), ER-II, ER-III, FP (FB 378), AT (FB 162), and EBF (FB 185). Authentic standards were purchased from Acmec Biochemical Technology (Shanghai, China), Aladdin Biochemical Technology (Shanghai, China), and Haoma Biotechnology (Guangzhou, China), with purities > 95%. The full names, abbreviations, CAS numbers, chemical structures, and key physicochemical properties of the 13 FB standards are detailed in Table A1. Acetonitrile (ACN), dichloromethane (DCM), tetrahydrofuran (THF), and methanol (MeOH) were obtained from Fisher Scientific (Fair Lawn, NJ, USA). Ultrapure water was prepared using a Milli-Q Integral system (MilliporeSigma, Billerica, MA, USA). SPE was performed using Captiva EMR-Lipid (3cc, Agilent Technologies, Santa Clara, CA, USA), HLB (3cc, Waters, Milford, MA, USA), florisil (Anple, Shanghai, China), and alumina (Sigma-Aldrich, St. Louis, MO, USA).

### 2.2. Study Population and Sample Collection

The plasma samples of adults were obtained from 10 volunteers randomly recruited in 2020 at Zhujiang Hospital of Southern Medical University. Approximately 10 mL of blood was collected from each volunteer using heparinized vacuum blood collection tubes. Each blood sample was immediately centrifuged at 4000 rpm for 10 min at 4 °C to separate the plasma from the red blood cells. The resulting plasma samples were stored at −80 °C before chemical analysis. The research content of this study was approved by both the ethics committee of Jinan University and the hospital, and informed consent was obtained from each participant.

### 2.3. Standard Solutions

Due to differences in analyte solubility, the standards of 13 analytes were dissolved in various solvents to prepare individual standard stock solutions at a concentration of 100 mg/L. Specifically, OB-2, OB-1, OB, and KSN were dissolved in THF; KCB, PF, FP, ER-I, ER-II, and ER-III in DCM; and SWN, EBF, and AT in ACN. Each individual stock solution was then diluted with ACN to obtain a mixed working solution at a concentration of 1 mg/L. Prior to use, the mixed stock solution was progressively diluted with ACN to prepare solvent calibration solutions with concentrations ranging from 0.01 to 50 ng/mL.

### 2.4. Sample Preparation

In this study, three different sample preparation procedures for human plasma were proposed, primarily differing in their purification approaches. These include Florisil-alumina composite SPE, Oasis HLB SPE, and Captiva EMR-Lipid SPE. The specific preparation methods are described below.

Florisil-alumina composite SPE: Based on our previously established method for analyzing FBs in sludge [15], modifications were made as follows: A 0.5 mL plasma sample was placed in a 10 mL glass centrifuge tube, and 3 mL of DCM/ACN (3:7, *v*/*v*) along with 0.2 g of NaCl were added. Liquid–liquid extraction was performed by vortexing for 10 min and ultrasonication for 20 min. After centrifugation at 4 °C, the supernatant was transferred to another glass tube. The extraction was repeated three times, and the combined extracts were concentrated to 1 mL using nitrogen evaporation. A composite SPE column was prepared by sequentially packing 2 g of 5% water-deactivated Florisil and 2 g of 5% water-deactivated alkaline alumina from bottom to top. Prior to sample loading, the column was conditioned with 10 mL of DCM/ACN (3:7, *v*/*v*). After loading the concentrated extract, the column was eluted with 15 mL of DCM/ACN (3:7, *v*/*v*). The eluate was collected, concentrated to 0.5 mL using nitrogen evaporation, and then filtered through a 0.22 μm polytetrafluoroethylene (PTFE) filter for instrumental analysis.

Oasis HLB SPE: A 0.5 mL plasma sample was introduced into a 10 mL glass centrifuge tube, and 2 mL of ACN was added. The mixture was vortexed for 15 min and centrifuged to precipitate proteins. The supernatant was collected and concentrated to approximately 0.5 mL under a gentle stream of nitrogen. The concentrated supernatant was diluted with 2 mL of ultrapure water. An HLB column was preconditioned sequentially with 3 mL of MeOH and 3 mL of ultrapure water. The water-diluted sample was loaded onto the column, followed by a wash with 3 mL of 25% MeOH in water. The target analytes were eluted with 6 mL of MeOH, and the eluate was collected. The eluate was concentrated to 0.5 mL under a nitrogen stream and filtered through a 0.22 μm PTFE filter prior to instrumental analysis.

Captiva EMR-Lipid SPE: A 0.5 mL plasma sample was combined with 2 mL of ACN in a 10 mL glass centrifuge tube. The mixture was vortexed for 10 min and then centrifuged at 4 °C for 5 min to ensure complete protein separation. The resulting solution was passed through a Captiva EMR-Lipid column, and, subsequently, an additional 3 mL of 80% ACN in water was added to the column. All the eluted solutions were collected, totaling approximately 5.5 mL. To induce phase separation, 0.5 g of NaCl was added to the eluted solution. The upper ACN phase was isolated and concentrated to 0.5 mL using nitrogen evaporation. The concentrated solution was filtered through a 0.22 μm PTFE filter before instrument analysis.

### 2.5. Instrument Analysis

Quantitative analysis of 13 FBs was conducted using an ExionLC AC ultrafast liquid chromatograph coupled with a Triple Quad 5500 mass spectrometer (AB SCIEX, Framingham, MA, USA). Chromatographic separation was performed on an XBridge BEH C18 column (2.5 μm, 2.1 mm × 100 mm) at 40 °C (Waters Corporation, Milford, MA, USA), with the mobile phase consisting of (A) water containing 0.1% formic acid and (B) ACN at a flow rate of 0.3 mL/min. The elution gradient was programmed as follows: 0–1 min, 10% B; 8–15 min, 90% B; 15.1–18 min, 100% B; and 18.1–23 min, 10% B. The injection volume was set to 5 μL. The mass spectrometer operated in positive electrospray ionization (ESI) mode and multiple reaction monitoring (MRM) mode. The spray voltage was configured at 5500 V, the ion source temperature was adjusted to 500 °C, the curtain gas pressure was set to 40 psi, and both the spray gas and auxiliary heating gas pressures were maintained at 40 psi. The optimized MS/MS parameters for all analytes are listed in Table A2.

### 2.6. Validation Procedure

The proposed method was validated in terms of linearity, method detection limit (MDL), method quantitation limit (MQL), matrix effect (ME), truthfulness, and precision. Matrix-matched calibration curves were prepared using blank matrix solutions across a concentration range of 0.01–50 ng/mL. The R^2^ value of the matrix calibration curve was used to evaluate the linearity of the method. The MDL was defined as the analyte concentration corresponding to a signal-to-noise ratio of 3, while the MQL and the lower limit of the linear range were defined as 10. Ultrapure water was used as a procedural blank, replacing human plasma in the pretreatment process. When an analyte was detected in the procedural blank, its MDL and MQL were defined as 3 and 10 times the standard deviation of the procedural blank’s concentration level. The ME, calculated using the formula ME (%) = (slope of matrix-matched calibration curve/slope of solvent calibration curve) × 100%, was employed to assess the degree of ion suppression or enhancement by the matrix. The truthfulness of the method was verified through the recovery rate of plasma samples (*n* = 3) at a spiking level of 10 ng/mL and the procedural blank, and the precision of the method was validated by calculating the relative standard deviation of the recovery rates. The RSDs of the spiked recoveries for all compounds ranged from 1% to 11%, indicating the high precision of the method.

### 2.7. Data Analysis

Chromatographic peaks were integrated using Analyst 1.6.3 (AB SCIEX), and the peak areas were substituted into the calibration curve to calculate the concentrations of target analytes. Since ER-I, ER-II, and ER-III differ only in the position of the side chain on the benzene ring (e.g., adjacent, intermediate, para), they display identical parent and daughter ions in the mass spectra. Therefore, it is not feasible to separate the isomeric compounds through chromatography, and they were collectively quantified. Statistical analysis of the data was performed using SPSS 27 (IBM Corporation, Armonk, NY, USA), and concentrations below the MDL/MQL were replaced with half of the MDL/MQL values for statistics.

## 3. Results and Discussion

### 3.1. Optimization of Instrument Conditions

**Figure A1:**
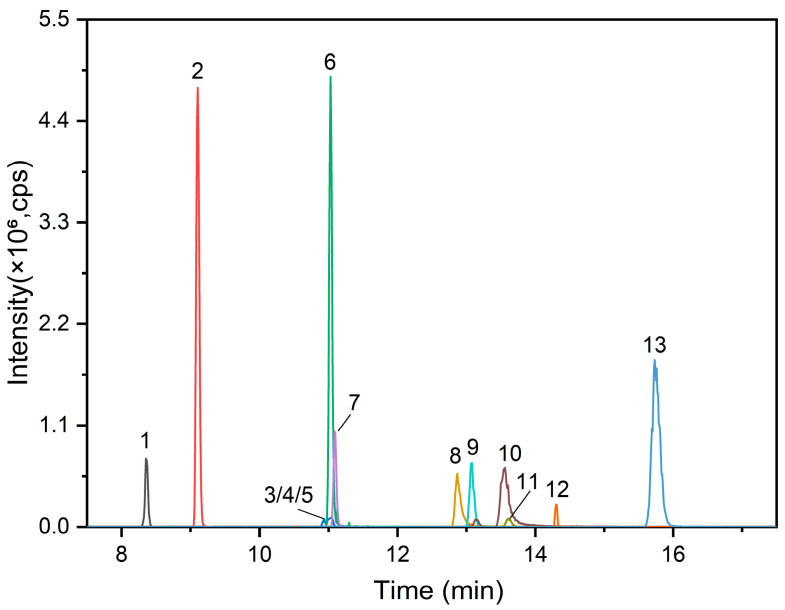
Total chromatograms of 13 target FBs. 1. AT, 2. SWN, 3. ER-I, 4. ER-II, 5. ER-III, 6. PF, 7. EBF, 8. OB-1, 9. KCB, 10. KSN, 11. FP, 12. OB-2, 13. OB.

In our previous research, systematic optimization was conducted on the chromatographic and mass spectrometric conditions for analyzing 13 FBs that were under investigation [16]. The chromatographic conditions encompassed the type of mobile phase, the categories and proportions of mobile phase additives, the type of chromatographic column, and the gradient elution profile. The mass spectrometric conditions involved ion source parameters (such as spray voltage, ion source temperature, spray gas, auxiliary heating gas, and curtain gas) and compound-specific parameters (including collision energy and declustering potential). Based on this established method, the present study further refined the gradient elution program for instrumental analysis. Total chromatograms of 13 target FBs are shown in Figure A1.

### 3.2. Optimization of Clean-Up Procedure

The Florisil-alumina composite SPE is a purification method successfully developed and applied by our team for the analysis of FBs in complex environmental samples, i.e., sludge. This method combines two classic polar adsorbents, Florisil and alkaline alumina, primarily designed to remove polar compounds from environmental samples [21], such as pigments and humic acids. However, due to its limited adsorption capacity for non-polar lipids, its application to human plasma samples may present certain limitations. The sorbent of HLB SPE cartridges is a hydrophilic–lipophilic balanced polymer, capable of retaining both non-polar and polar compounds [22]. As a result, gradient elution can be utilized to achieve the separation of interfering matrix components from target analytes. This feature makes HLB SPE widely applicable for the purification of human samples [23,24]. Captiva EMR-Lipid is a specialized SPE product designed for the removal of lipid interference. Its mechanism combines size exclusion and hydrophobic interactions between the sorbent and the long alkyl chain functional groups of lipids, enabling selective adsorption of lipids without significant loss of target analytes [25].

**Figure 1:**
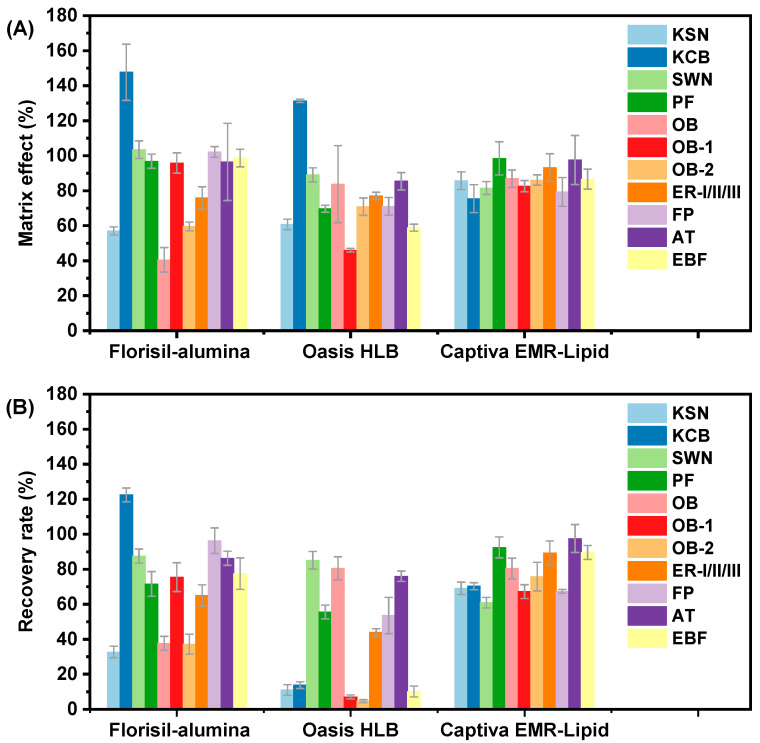
(**A**) Post-extraction spiked matrix effect and (**B**) sample spiked recovery rate of three candidate purification methods, both evaluated at a spiking level of 10 ng/mL.

Using the three purification methods described above as candidates for human plasma samples, the MEs of the samples processed by each method were rapidly evaluated through the post-extraction spike method (Figure 1A). Both the Florisil-alumina composite SPE and HLB SPE purification methods resulted in significant matrix suppression for KSN (ME < 60%) and matrix enhancement for KCB in plasma samples. Additionally, the Florisil-alumina composite SPE showed severe matrix suppression for OB and OB-2, while HLB SPE exhibited similar suppression for OB-1 and EBF. These results indicate that the purification efficiency of both methods is insufficient to meet the analytical requirements for target FBs in plasma samples. In contrast, after purification with EMR-Lipid SPE, the ME values for all target FBs in plasma samples fell within the range of 80–120%, indicating no significant matrix suppression or enhancement. We further compared the spiked recoveries of individual FBs in plasma samples after pretreatment using these three SPE purification procedures (Figure 1B). The spiked recoveries after purification with Florisil-alumina composite SPE exhibited results similar to its matrix effects, indicating no significant analyte loss during the pretreatment process; however, due to the presence of matrix effects, satisfactory spiked recoveries could not be achieved using this method. After pretreatment with HLB SPE, some FBs exhibited spiked recoveries below 20%, primarily due to matrix suppression and analyte loss during the washing step. This indicates that gradient elution on the HLB column cannot simultaneously achieve the separation of interfering substances from FBs. Purification with EMR-Lipid resulted in satisfactory recoveries for all target FBs in plasma samples, ranging from 61% to 98%. Combined with the results above, EMR-Lipid SPE ensures both the recovery of FBs and the removal of matrix interference, making it a suitable clean-up procedure for the analysis of FBs in plasma samples. The method combining EMR-Lipid SPE with LC-MS/MS analysis has the potential for qualitative and quantitative analysis of trace FBs in human plasma.

### 3.3. Method Validation

The applicability of the method combining EMR-Lipid SPE purification of plasma samples with LC-MS/MS analysis was validated from multiple perspectives, including linearity, MDL, MQL, procedural blank, ME, as well as the aforementioned spiked recovery and its RSDs (Table 1). The method exhibited good linearity within the investigated range of approximately 0.01–50 ng/mL, with R^2^ values greater than 0.992. The MDLs and MQLs ranged from 0.004 to 0.105 ng/mL and from 0.012 to 0.348 ng/mL, respectively. The sufficiently high sensitivity meets the requirements for the analysis of trace FBs in plasma. The MEs were further evaluated by comparing the slopes of the matrix-matched calibration curves and the solvent calibration curves. The results showed that the matrix effects ranged from 72% to 100%, once again indicating effective sample purification performance achieved with this method. In the procedural blanks, only trace levels of OB (mean: 0.017 ng/mL) and EBF (mean: 0.016 ng/mL) were detected. This result, together with the spiked recoveries achieved by this method, demonstrates its truthfulness. Therefore, this method can be regarded as a reliable approach for the qualitative and quantitative analysis of 13 FBs at trace levels in human plasma.

### 3.4. Analysis of FBs in Human Plasma Samples

The validated method combining EMR-Lipid SPE with LC-MS/MS analysis was applied to determine the concentrations of 13 FBs in 10 human plasma samples. Descriptive statistics of the measured concentrations are presented in Table 2. Among the 13 FBs, 10 were detected in human plasma samples, with total concentrations ranging from 0.221 to 0.684 ng/mL. The detection frequency of OB-1, EBF, and KSN was ≥50%. OB-1 was detected in all samples, with a median concentration of 0.061 ng/mL, and represented one of the most predominant FBs in plasma, accounting for 28.0% of the total 13 FBs. FP was another major FB in plasma, with an abundance of 28.7%. Despite being detected in only one sample (concentration: 0.405 ng/mL) due to the relatively high MQL, its presence remains non-negligible. The abundances of the remaining FBs followed the order of KSN (18.5%), ER-I/II/III (17.5%), EBF (2.8%), OB (2.6%), and SWN (1.8%). This distribution is consistent with the compositional characteristics of FBs observed in indoor environments from our previous investigations [16], suggesting that human exposure to FBs may be closely linked to indoor sources. These results further demonstrate the applicability and reliability of this analytical method and highlight the co-occurrence of multiple FBs in human blood, emphasizing the need for further attention to the potential health risks of exposure to these chemicals.

## 4. Conclusions

In this study, a method for analyzing multiple trace-level FBs in human plasma was developed and validated for the first time. The method demonstrated good linearity, sensitivity, accuracy, and precision for both qualitative and quantitative analysis, addressing the current lack of an effective approach for assessing internal human exposure levels to FBs, which are considered new pollutants. Additionally, the application of this method to actual human plasma samples resulted in the detection of 10 FBs, with some compounds exhibiting high detection rates, suggesting that these pollutants may be widely present in the human body. Further research with larger sample sizes and detailed exposure assessments is recommended to better understand the sources, distribution, and health effects of FBs in human populations. Moreover, future studies could explore the metabolic pathways and toxicological implications of these compounds to assess their long-term impact on human health.

## Figures and Tables

**Table 1:** Linear ranges, R^2^ values, MDLs, MQLs, procedural blanks, matrix effects (MEs), recovery rates, and relative standard deviations (RSDs) of 13 FBs in human plasma achieved using EMR-Lipid SPE and LC-MS/MS analysis.

Analyte	Linear Range (ng/mL)	R^2^	MDL (ng/mL)	MQL (ng/mL)	Blank (ng/mL)	ME (%)	Recovery Rate (%)	RSD (%)
KSN	0.070–50	0.998	0.021	0.070	nd ^1^	81	69	5
KCB	0.233–50	0.995	0.070	0.233	nd	72	70	3
SWN	0.019–50	0.995	0.006	0.019	nd	73	61	5
PF	0.017–50	0.998	0.005	0.017	nd	100	92	6
OB	0.011–50	0.996	0.007	0.022	0.017	83	80	7
OB-1	0.024–50	0.992	0.007	0.024	nd	79	67	6
OB-2	0.261–50	0.992	0.078	0.261	nd	87	76	11
ER-I/II/III	0.116–50	0.995	0.035	0.116	nd	91	89	8
FP	0.348–50	0.992	0.105	0.348	nd	79	67	1
AT	0.012–50	0.999	0.004	0.012	nd	94	98	8
EBF	0.015–50	0.997	0.005	0.017	0.016	85	90	4

^1^ nd: not detectable.

**Table 2:** Descriptive statistics of concentrations (ng/mL) of 13 FBs in 10 human plasma samples.

Analyte	DF ^1^ (%)	GM ^2^	Median	Mean	Range	AP ^3^ (%)
OB-1	100	0.058	0.061	0.069	<MQL to 0.132	28.0
EBF	50	<MQL	<MQL	<MQL	<MQL to 0.021	2.8
KSN	50	<MQL	<MQL	<MQL	<MQL to 0.151	18.5
ER-I/II/III	40	<MQL	<MQL	<MQL	<MQL to 0.131	17.5
OB	20	<MQL	<MQL	<MQL	<MQL to 0.033	2.6
SWN	10	<MQL	<MQL	<MQL	<MQL to 0.023	1.8
FP	10	<MQL	<MQL	<MQL	<MQL to 0.405	28.7
AT	10	<MQL	<MQL	<MQL	<MQL	-
KCB	0	<MQL	<MQL	<MQL	<MQL	-
PF	0	<MQL	<MQL	<MQL	<MQL	-
OB-2	0	<MQL	<MQL	<MQL	<MQL	-
∑_13_FBs	-	0.333	0.320	0.354	0.221 to 0.684	100

^1^ DF: detection frequency. ^2^ GM: geometric mean. ^3^ AP: average proportion.

## Data Availability

The original contributions presented in this study are included in the article. Further inquiries can be directed to the corresponding author.

## Appendix A

**Table A1:** Chemical names, abbreviations, CAS numbers, molecular formulas, chemical structures, and key physicochemical properties of 13 commonly used FBs.

Chemical Name	Abbreviation	CAS NO.	Molecular Formula	Chemical Structure	Log*K*_ow_
4-(2-Benzoxazolyl)-4′-(5-methyl-2-benzoxazolyl)stilbene	KSN; FB368	5242-49-9	C_29_H_20_N_2_O_2_		8.05
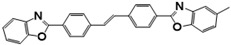
1,4-Bis(2-benzoxazolyl)naphthalene	KCB; FB 367	5089-22-5	C_24_H_14_N_2_O_2_		6.15
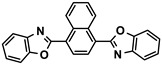
7-Diethylamino-4-methylcoumarin	SWN; FB 52; FB 140	91-44-1	C_14_H_17_NO_2_		3.22
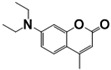
1,2-Bis(5-methyl-2-benzoxazolyl)ethylene	PF; DT; FB 135	1041-00-5	C_18_H_14_N_2_O_2_		5.07
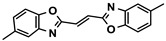
2,5-Bis(5-*tert*-butyl-2-benzoxazolyl)thiophene	OB; FB 184	7128-64-5	C_26_H_26_N_2_O_2_S		8.61
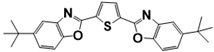
4,4′-Bis(2-benzoxazolyl)stilbene	OB-1; FB 393	1533-45-5	C_28_H_18_N_2_O_2_		7.5
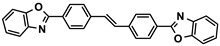
4,4′-Bis(5-methyl-2-benzoxazolyl)stilbene	OB-2	2397-00-4	C_30_H_22_N_2_O_2_		8.6
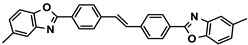
1,4-Bis(2-cyanostyryl)benzene	ER-I; FB 199	13001-39-3	C_24_H_16_N_2_		6.15
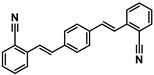
1-(2-Cyanostyryl)-4-(4-cyanostyryl)benzene	ER-II	13001-38-2	C_24_H_16_N_2_		6.15
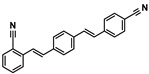
1,4-Bis(4-cyanostyryl)benzene	ER-III	13001-40-6	C_24_H_16_N_2_		6.15
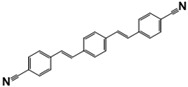
4,4′-Bis(2-methoxystyryl)biphenyl	FP; FB 378	40470-68-6	C_30_H_26_O_2_		8.98
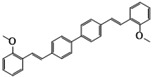
4-Methoxy-N-methyl-1,8-naphthalimide	AT; FB 162	3271-05-4	C_14_ H_11_NO_3_		2.28
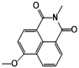
2,2′-(2,5-Thiophenediyl)bis-benzoxazole	EBF; FB 185	2866-43-5	C_18_H_10_N_2_O_2_S		4.79
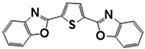

**Table A2:** Optimized ESI (+)-MS/MS parameters for 13 FBs.

Analyte	Precursor Ion	Product Ion	CE ^1^ (eV)	DP ^2^ (V)
KSN	429.1	221.3 * 321.1	52 54	30 30
KCB	363.1	65 * 269.2	78 56	220 220
SWN	232.1	160.1 * 188.1	48 40	161 188
PF	291.1	107 * 77.1	37 73	210 210
OB	431.2	385.1 * 399.2	80 75	86 86
OB-1	415.1	415.1 * 321.2	50 55	30 30
OB-2	443.3	335.2 * 336.2	55 49	30 30
ER-I/II/III	331.1	315.9 * 217.1	33 40	100 100
FP	419.2	119.1 * 91.1	37 57	80 80
AT	242	127.2 * 185	45 28	182 183
EBF	319.1	65.1 * 226	65 46	75 76

* Quantitative ion. ^1^ CE: collision energy. ^2^ DP: declustering potential.
